# Impact of body mass index on outcomes in patients undergoing transfemoral transcatheter aortic valve implantation

**DOI:** 10.1016/j.xjon.2021.03.012

**Published:** 2021-03-23

**Authors:** Astrid C. van Nieuwkerk, Raquel B. Santos, Samantha Sartori, Ander Regueiro, Didier Tchétché, Roxana Mehran, Ronak Delewi, CENTER collaboration, Flavio S. De Brito, Flavio Tarasoutchi, Marco Barbanti, Ran Kornowski, Katia Orvin, Azeem Latib, Matteo Pagnesi, Augusto D'Onofrio, Giuseppe Tarantini, Flavio Ribichini, Mattia Lunardi, Jan Baan, Jan Tijssen, José P.S. Henriques, Francisco Ten, Nicolas Dumonteil, Angie Ghattas, Paola D'Errigo, Juan Manuel Nogales, Thomas Modine, George Dangas

**Affiliations:** a Department of Clinical and Experimental Cardiology, Heart Center, Amsterdam UMC, University of Amsterdam, Amsterdam, The Netherlands; b Department of Cardiology, Centro Hospitalar Universitário do Porto, Porto, Portugal; c The Zena and Michael A. Wiener Cardiovascular Institute, Icahn School of Medicine at Mount Sinai, New York, NY; d Servicio de Cardiología, Hospital Clínic, Barcelona, Spain; e Clinique Pasteur, Toulouse, France

**Keywords:** transcatheter aortic valve implantation, aortic valve stenosis, obesity, body mass index, underweight, BE, balloon expandable, BMI, body mass index, CENTER, Cerebrovascular Events in Patients Undergoing Transcatheter Aortic Valve Implantation, SE, self-expandable, TAVI, transcatheter aortic valve implantation, VARC 2, Second Valve Academic Research Consortium

## Abstract

**Objective:**

This study sought to investigate the effect of body mass index on outcomes in patients with severe aortic valve stenosis undergoing transcatheter aortic valve implantation.

**Methods:**

A total of 12,381 patients undergoing transfemoral transcatheter aortic valve implantation were divided into body mass index categories: underweight (<18.5 kg/m^2^), normal weight (18.5-24.9 kg/m^2^), overweight (25.0-29.9 kg/m^2^), and obesity (>30 kg/m^2^). Primary endpoints were differences in 30-day and 1-year all-cause mortality. Secondary endpoints included all other clinical endpoints such as stroke. Univariate and multivariate odds ratios were calculated using logistic and cox regression analyses.

**Results:**

Two percent (n = 205) of patients were underweight, 29% (n = 3564) were normal weight, 44% (n = 5460) were overweight, and 25% (n = 3152) were obese. Thirty-day mortality was lower in overweight (5.3%, odds ratio, 0.73; 95% confidence interval, 0.61-0.88; *P* = .001) and obese patients (5.2%, odds ratio, 0.74; 95% confidence interval, 0.60-0.92; *P* = .006), but higher in underweight (9.8%, odds ratio, 1.51; 95% confidence interval, 0.92-2.47; *P* = .010) as compared to normal weight patients (6.9%). After multivariate adjustment, 30-day mortality was not significantly different across body mass index categories. However, 1-year mortality was higher in underweight patients (hazard ratio, 1.52; 95% confidence interval, 1.10-2.09; *P* = .011). Stroke rates were comparable between body mass index groups.

**Conclusions:**

For overweight and obese patients with severe aortic valve stenosis undergoing transcatheter aortic valve implantation, there was no 30-day difference in mortality compared with patients with normal weight. However, underweight patients showed higher rates of 1-year mortality after transcatheter aortic valve implantation.

## Graphical abstract

Mortality outcomes per body mass index (BMI) category in patients undergoing transcatheter aortic valve implantation (TAVI). *OR*, Odds ratio; *HR*, hazard ratio compared with normal-weight patients.

**Figure:**
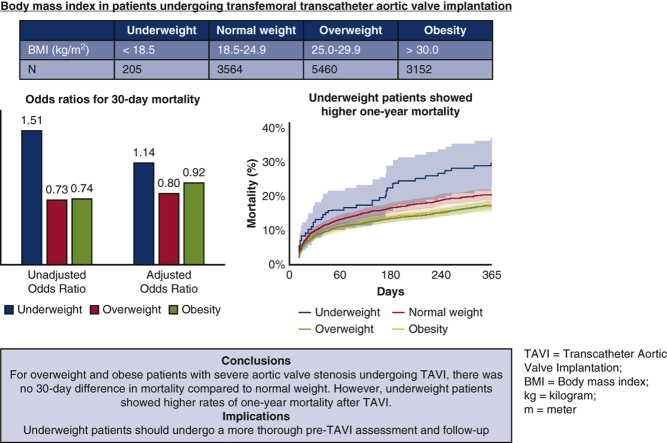

**Figure:**
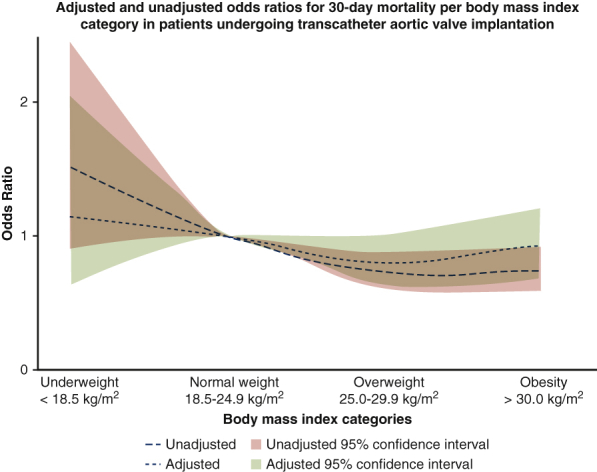
Odds ratios for 30-day mortality per body mass index category in patients undergoing TAVI.

**Central Message**

Overweight and obese patients with severe aortic valve stenosis undergoing TAVI showed no 30-day difference in mortality as compared to normal weight patients. However, underweight patients had higher rates of 1-year mortality.

**Perspective**

Underweight is an important predictor for 1-year mortality rates after TAVI. This warrants a more thorough assessment of these patients, including adjustments to improve this frailty and a closer follow-up.

See Commentaries on pages 37 and 39.

Transcatheter aortic valve implantation (TAVI) is a treatment option for patients with severe symptomatic aortic valve stenosis. Its indication has expanded from inoperable to low risk patients.^1^, ^2^, ^3^, ^4^, ^5^, ^6^

Obesity is an epidemic associated with the metabolic syndrome, including diabetes mellitus, dyslipidemia, obstructive sleep apnea, and increased risk of developing cardiovascular diseases.^7^ Obesity reduces life expectancy in general and increases the challenge of surgical procedures, with longer operation times and postoperative complications, such as wound infections.^8^^,^^9^ Paradoxically, overweight and obesity have sometimes been associated with improved survival in patients with cardiovascular conditions, particularly after surgical procedures such as surgical aortic valve replacement and coronary artery bypass grafting.^10^, ^11^, ^12^, ^13^, ^14^, ^15^ On the other hand, in elderly patients, low weight is frequently associated with frailty and cardiac cachexia, leading to worse outcomes after invasive procedures.^16^^,^^17^ Data on influence of body mass index (BMI) on complication rates and outcomes after TAVI are scarce.

In this study, we merged patient-level data from several global registries and prospective studies for >12,000 patients undergoing transfemoral TAVI.^18^ The aim of this analysis was to assess the effect of BMI on the outcomes after TAVI.

## Methods

### Study Design and Patient Sample

The Cerebrovascular Events in Patients Undergoing Transcatheter Aortic Valve Implantation (CENTER) study is an international collaboration, including patients with severe aortic valve stenosis undergoing transfemoral TAVI with balloon-expandable devices (BE) (Edwards lifesciences Inc, Irvine Calif) or self-expandable devices (SE) (Medtronic Inc, Minneapolis, Minn). The study is registered at CinicalTrials.gov (NCT03588247). A more detailed description on study design, eligibility criteria, systematic search methodology, and data collection has been reported elsewhere.^19^ Briefly, the CENTER collaboration includes data from 3 national registries, 2 multicenter prospective registries, 4 single-center prospective registries, and 1 randomized clinical trial identified through a systematic online search on PubMed. Therefore, the collaboration comprises a global patient sample with patients treated in the United States of America, Brazil, Israel, and Europe. All studies were conducted according to the Declaration of Helsinki and all patients provided written informed consent at each participating center. Collaborators provided a dedicated database with baseline patient characteristics, echocardiography data, procedural information, and long-term clinical follow-up data. In total, 12,381 patients undergoing transfemoral TAVI between 2007 and 2018 with BE or SE valves were included in the CENTER study. BMI was calculated as weight in kilograms divided by the square of height in meters. Categorization of the BMI was adopted from the World Health Organization and National Institutes of Health and defined as underweight (<18.5 kg/m^2^), normal weight (18.5-24.9 kg/m^2^), overweight (25.0-29.9 kg/m^2^), and obese (≥30.0 kg/m^2^). For a more detailed analysis of outcomes in the obesity category, it was subsequently subdivided in obese (30.0-34.9 kg/m^2^), very obese (35.0-39.9 kg/m^2^), and severely obese (≥40.0 kg/m^2^).

### Study Endpoints

The primary endpoints of this analysis were differences in 30-day and 1-year all-cause mortality after TAVI between BMI categories (underweight: <18.5, normal weight: 18.5-24.9, overweight: 25.0-29.9, and obese: ≥30.0 kg/m^2^). Secondary outcomes included incidence of in-hospital mortality, stroke, myocardial infarction, life-threatening or major bleeding, requirement for permanent pacemaker and new-onset atrial fibrillation, as well as 30-day and 1-year stroke rates. All outcomes were reported as defined by the standardized definitions from the Second Valve Academic Research Consortium (VARC2).^20^ Outcomes were adjudicated by the adjudication committee in 6 of the 10 participating centers.^18^

### Statistical Analysis

We applied multiple imputation methods to estimate missing data in baseline medical history. The imputation procedure and multivariate regression models were performed according to the Rubin's protocol under the assumption that missing data are missing at random. Baseline values of continuous variables were tested for normal distribution and reported as mean ± standard deviation or median with interquartile range (IQR, 25th-75th percentile) as appropriate. Afterwards, the 1-way analysis of variance for continuous covariates or Kruskal-Wallis test was used to determine differences between BMI categories. Baseline categorical variables were presented as frequencies and percentages, differences between groups were tested with the Pearson χ^2^ test. The difference in incidence of in-hospital and 30-day outcomes for underweight, normal weight, overweight, and obese patients were reported. The normal weight group was used as reference group and we estimated odds ratios (ORs) and 95% confidence intervals (CIs) for 30-day mortality in the other BMI categories using logistic regression. Furthermore, baseline patient characteristics were explored as predictors of 30-day mortality, using logistic regression methods. Each potential predictor, dichotomous or continuous, was tested in a univariate model: those variables known from the literature or with *P* < .10 were combined in a multivariate model and presented as OR and 95% CI. Cox proportional hazards regression curves were made to assess cumulative 1-year mortality of the BMI groups and hazard ratios (HR) were reported. We included a frailty term to adjust for possible confounding. A frailty term (γ) assessed random effects and heterogeneity between trials and because of unmeasured factors. We corrected for possible clustering with generalized least squares model because data were collected from different centers that could influence outcomes. Sensitivity analyses were performed using *E* values, which indicate robustness of an association to potential confounding.^21^ All statistical tests were 2-tailed. Because 10 secondary outcomes were tested, Bonferroni adjustment for multiple testing was applied. All calculations were generated by SPSS software version 26.0 for Windows (IBM-SPSS, Inc, Armonk, NY).

## Results

### Patient Population

A total of 12,381 patients were included. Median age was 83 years (IQR, 80-88 years), 58% were women, and mean BMI was 27.2 ± 4.9 kg/m^2^. Two percent of patients (n = 205) were underweight, 29% (n = 3553) were normal weight, 44% (n = 5470) were overweight, and 25% (n = 3150) were obese. Baseline characteristics of patients according to BMI category are presented in Table 1. Cardiovascular risk factors such as hypertension, diabetes mellitus and dyslipidemia increased progressively with increase of BMI category. Low glomerular filtration rate <30 mL/min/1.73 m^2^ was inversely correlated with BMI category: from 29% in underweight to 8% in obese patients (*P* < .001). Mortality risk prediction scores, Logistic European System for Cardiac Operative Risk Evaluation, European System for Cardiac Operative Risk Evaluation II, and Society of Thoracic Surgeons Predicted Risk of Mortality were lower with increase in BMI category.

**Table 1:** Baseline patient characteristics of patients undergoing transcatheter aortic valve implantation according to body mass index category

	BMI category					*P* value
	Total (N = 12,381)	<18.5 kg/m^2^ (n = 205)	18.5-24.9 kg/m^2^ (n = 3564)	25-29.9 kg/m^2^ (n = 5460)	≥ 30 kg/m^2^ (n = 3152)	
Demographics						
Age (y)	83 (78-86)	84 (80-88)	84 (80-87)	83 (79-86)	81 (77-85)	<.001
Female sex	7105 (58)	170 (83)	2147 (60)	2823 (52)	1965 (63)	<.001
Medical history						
Stroke or TIA	1288 (10)	23 (11)	354 (10)	598 (11)	313 (10)	.35
Myocardial infarction	1666 (14)	24 (12)	456 (13)	786 (14)	400 (13)	.07
Previous PCI	2654 (22)	26 (13)	743 (21)	1205 (22)	680 (22)	.01
Previous CABG	1469 (12)	7 (3)	395 (11)	699 (13)	368 (12)	<.001
Hypertension	9714 (79)	134 (65)	2540 (72)	4356 (80)	2684 (86)	<.001
Diabetes mellitus	3867 (31)	32 (16)	808 (23)	1664 (31)	1363 (43)	<.001
Dyslipidemia	6778 (55)	83 (41)	1697 (48)	3064 (56)	1934 (62)	<.001
Peripheral vascular disease	1814 (15)	31 (15)	553 (16)	846 (16)	373 (12)	<.001
Coronary artery disease	5070 (41)	75 (37)	1506 (42)	2298 (42)	1191 (38)	<.001
Atrial fibrillation	3345 (27)	50 (24)	959 (27)	1493 (27)	843 (27)	.79
GFR <30 mL/min/1.73 m^2^	1682 (14)	59 (29)	691 (20)	674 (12)	256 (8)	<.001
Risk scores						
Logistic EuroSCORE (%)	15.0 (9.5-23.0)	17.4 (11.8-29.1)	16.0 (10.1-25.6)	15.0 (9.5-22.6)	13.2 (8.8-20.7)	<.001
EuroSCORE II (%)	4.0 (2.3-6.9)	4.8 (3.3-7.9)	4.1 (2.6-7.1)	3.9 (2.3-6.9)	3.7 (2.1-6.0)	<.001
STS-PROM (%)	6.4 (4.0-13.0)	9.6 (5.2-18.3)	7.5 (4.6-14.5)	6.0 (3.8-12.2)	5.8 (3.4-11.2)	<.001
Echocardiographic aortic characteristics						
Max gradient (mm Hg)	79 ± 23	84 ± 26	80 ± 24	79 ± 23	79 ± 23	.02
Mean gradient (mm Hg)	51 ± 17	53 ± 20	51 ± 18	51 ± 18	50 ± 16	.002
Aortic valve area (cm^2^)	0.7 ± 0.2	0.6 ± 0.2	0.6 ± 0.2	0.7 ± 0.2	0.7 ± 0.2	<.001
Device type						
Balloon-expandable valve	6226 (50)	96 (47)	1844 (52)	2785 (51)	1501 (48)	.004
Self-expandable valve	6131 (50)	109 (53)	1708 (48)	2675 (49)	1639 (52)	.004

Values are presented as median (interquartile range), n (%), or mean ± standard deviation. *BMI*, Body mass index; *TIA*, transient ischemic attack; *PCI*, percutaneous coronary intervention; *CABG*, coronary artery bypass grafting; *GFR*, glomerular filtration rate; *EuroSCORE*, European System for Cardiac Operative Risk Evaluation; *STS-PROM*, Society of Thoracic Surgeons Predicted Risk of Mortality.

### Mortality

**Figure 1:**
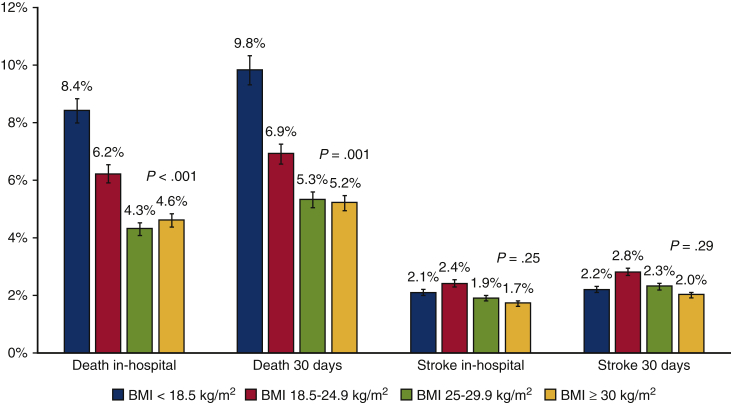
Incidence of 30-day and in-hospital all-cause mortality and stroke according to body mass index (*BMI*) categories: *blue* indicates underweight, *red* indicates normal weight, *green* indicates overweight, and *yellow* indicates obesity.

Mean follow-up was 464 ± 536 days and median follow-up was 365 days (IQR, 56-667 days). In-hospital outcomes were 98% complete and 30-day outcomes were complete in 91% of patients. Missing data per BMI category are displayed in Table E1. In-hospital and 30-day outcomes according to BMI categories are summarized in Table 2 and Figure 1. Thirty-day mortality was 6.9% in normal weight patients (reference category). Thirty-day mortality was highest in underweight patients (9.8%, OR, 1.51; 95% CI, 0.92-2.47; *P* = .010). Mortality was 5.3% in overweight and 5.2% in obese patients (OR, 0.73; 95% CI, 0.61-0.88; *P* = .001 and OR, 0.74; 95% CI, 0.60-0.92; *P* = .006, respectively).

**Table 2:** Unadjusted in-hospital, 30-day, and 1-year clinical outcomes according to body mass index category

Outcome	BMI category			
	<18.5 kg/m^2^ (n = 205)	18.5-24.9 kg/m^2^ (n = 3564)	25-29.9 kg/m^2^ (n = 5460)	>30.0 kg/m^2^ (n = 3152)
During hospital admission				
Mortality	14 (8.4)	184 (6.2)	205 (4.3)	134 (4.6)
Stroke	4 (2.1)	79 (2.4)	96 (1.9)	51 (1.7)
Myocardial infarction	0 (0.0)	27 (0.8)	37 (0.7)	20 (0.7)
Major or life-threatening bleeding	10 (5.9)	191 (6.3)	284 (6.0)	123 (4.4)
New onset atrial fibrillation	8 (6.1)	110 (6.0)	105 (4.7)	57 (5.8)
Permanent pacemaker implantation	18 (9.8)	338 (12.3)	697 (15.0)	421 (14.8)
At 30 d				
Mortality	18 (9.8)	218 (6.9)	254 (5.3)	144 (5.2)
Stroke	4 (2.2)	88 (2.8)	112 (2.3)	56 (2.0)
At 1 y				
Mortality	41 (28.7)	458 (18.9)	538 (15.5)	325 (15.7)
Stroke	10 (6.9)	137 (5.6)	186 (5.3)	90 (4.3)

Values are presented as n (%). Data about in-hospital mortality was complete in 98%; in-hospital stroke in 98%; myocardial infarction in 94%; major bleeding in 91%; new-onset atrial fibrillation in 57%; pacemaker implantation in 92%; 30-day mortality in 91%; 30-day stroke in 91%; 1-year mortality in 66%; and 1-year stroke in 66%. *BMI*, Body mass index.

**Figure 2:**
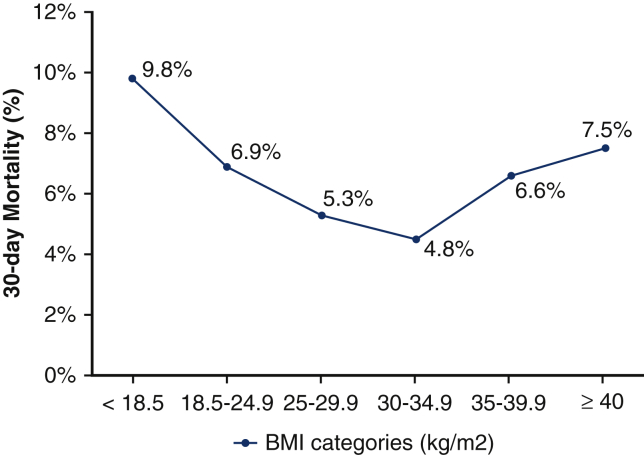
Thirty-day all-cause mortality according to body mass index (*BMI*) categories: <18.5 is underweight, 18.5 to 24.9 is normal weight, 25.0 to 29.9 is overweight, 30.0 to 34.9 is obese, 35.0 to 39.9 is very obese, and ≥40 is severely obese.

Obese patients were further subdivided into obese (BMI 30.0-34.9), very obese (BMI 35.0-39.9), and severely obese patients (BMI ≥ 40.0). Thirty-day mortality was 4.8% in obese, 6.6% in very obese, and 7.5% in severely obese patients (Figure 2). Very and severely obese patients did not show a survival benefit compared with normal weight patients (OR, 0.95; 95% CI, 0.67-1.35; *P* = .79 and OR, 1.09; 95% CI, 0.62-1.84; *P* = .76, respectively).

**Figure 3:**
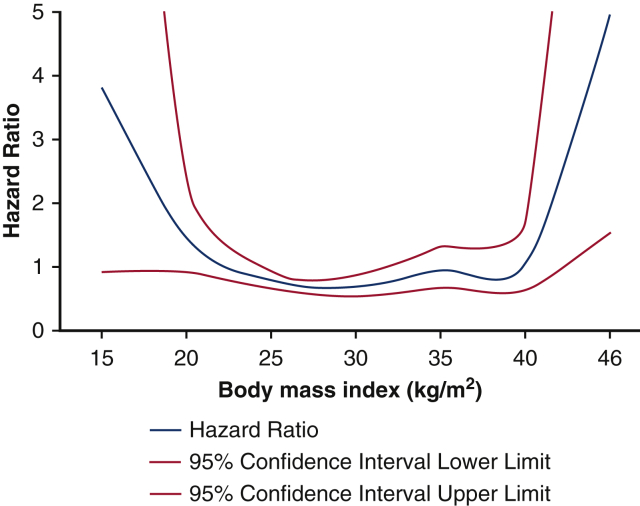
Association between body mass index (BMI) and hazard ratios for 30-day mortality compared with median BMI as a reference value. *Red lines* present upper and lower limits of the 95% confidence interval.

To further gain insight into the association of BMI and 30-day mortality, we presented HRs of BMI using the median BMI of 27.0 as reference value in Figure 3. There was no difference in 30-day mortality for BMI categories between different centers where TAVI was performed (*z* = 0.71; *P* = .48). Thirty-day mortality did not differ between BE valves compared with SE valves in each BMI category (Table E2). In a multivariate analysis, BMI categories were not independent predictors of 30-day mortality (Table 3).

**Table 3:** In-hospital and 30-day outcomes according to body mass index category

Category	Unadjusted odds ratio (95% confidence interval)	*P* value	*E* value	Adjusted odds ratio (95% confidence interval)	*P* value	*E* value
In hospital mortality						
Underweight	1.49 (0.87-2.53)	.14	2.43	1.03 (0.54-1.97)	.92	1.21
Normal weight	Reference	–		Reference	–	
Overweight	0.76 (0.63-0.93)	.007	1.96	0.83 (0.64-1.07)	.15	1.70
Obese	0.80 (0.64-1.00)	.048	1.81	0.94 (0.70-1.28)	.70	1.32
In hospital stroke						
Underweight	0.82 (0.30-2.52)	.70	1.74	0.66 (0.20-2.14)	.49	2.40
Normal weight	Reference	–		Reference	–	
Overweight	0.82 (0.61-1.10)	.18	1.74	0.88 (0.62-1.25)	.47	1.53
Obese	0.76 (0.54-1.08)	.12	1.96	0.68 (0.43-1.08)	.11	2.30
Myocardial infarction						
Underweight	0.00	1.00	-	0.00	1.00	-
Normal weight	Reference	-		Reference	–	
Overweight	0.90 (0.55-1.47)	.68	1.46	0.94 (0.49-1.79)	.84	1.32
Obese	0.84 (0.48-1.48)	.55	1.67	1.11 (0.54-2.27)	.78	1.46
Major- or life-threatening bleeding						
Underweight	0.92 (0.51-1.68)	.79	1.39	0.92 (0.49-1.75)	.81	1.39
Normal weight	Reference	–		Reference	–	
Overweight	0.91 (0.76-1.09)	.32	1.43	0.74 (0.59-0.94)	.012	1.32
Obese	0.65 (0.52-0.81)	<.001	2.45	0.62 (0.46-0.82)	.001	2.61
New-onset atrial fibrillation						
Underweight	1.11 (0.52-2.35)	.79	1.46	1.36 (0.63-2.93)	.44	2.06
Normal weight	Reference	–		Reference	–	
Overweight	0.88 (0.65-1.17)	.36	1.53	0.92 (0.66-1.29)	.64	1.39
Obese	1.11 (0.79-1.57)	.56	1.46	1.26 (0.85-1.88)	.26	1.83
Permanent pacemaker implantation						
Underweight	0.77 (0.48-1.25)	.29	1.92	0.68 (0.37-1.25)	.21	2.30
Normal weight	Reference	–		Reference	–	
Overweight	1.26 (1.10-1.44)	.001	1.83	1.30 (1.10-1.53)	.002	1.92
Obese	1.27 (1.10-1.48)	.001	1.86	1.30 (1.07-1.58)	.009	1.92
30-d mortality						
Underweight	1.51 (0.92-2.47)	.10	2.39	1.14 (0.63-2.04)	.67	1.54
Normal weight	Reference	–		Reference	–	
Overweight	0.73 (0.61-0.88)	.001	2.08	0.80 (0.63-1.01)	.06	1.81
Obese	0.74 (0.60-0.92)	.006	2.04	0.92 (0.68-1.21)	.52	1.39
30-d stroke						
Underweight	0.91 (0.37-2.26)	.84	1.39	0.82 (0.30-2.30)	.71	1.74
Normal weight	Reference	–		Reference	–	
Overweight	0.86 (0.66-1.13)	.27	1.60	0.92 (0.66-1.27)	.60	1.39
Obese	0.74 (0.53-1.02)	.06	2.04	0.68 (0.44-1.05)	.08	2.30

The multivariate model included age, sex, history of percutaneous coronary intervention, history of coronary artery bypass graft, diabetes mellitus, hypertension, dyslipidemia, glomerular filtration rate <30 mL/min/1.73 m^2^, valve type, mean aortic valve gradient, and logistic European System for Cardiac Operative Risk Evaluation and Society of Thoracic Surgeons Predicted Risk of Mortality.

**Figure 4:**
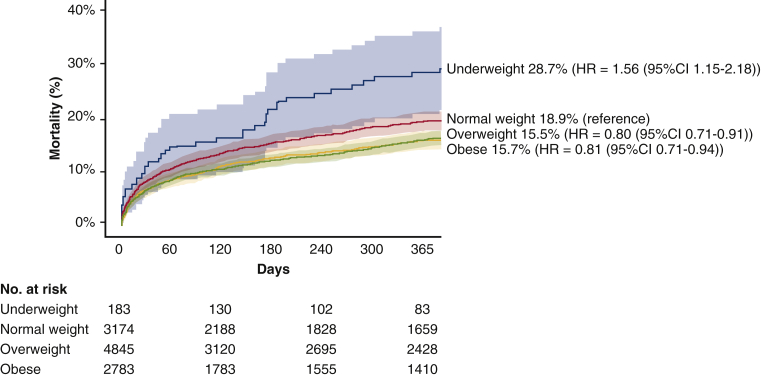
Kaplan-Meier time-to-event curves and 95% confidence intervals (*CIs*) for all-cause mortality according to 4 body mass index (BMI) categories up to 1 year after transcatheter aortic valve implantation. *HR*, Hazard ratio for 1-year mortality.

One-year mortality was lowest in overweight (15.5%, HR, 0.80; 95% CI, 0.71-0.91; *P* < .001) and obese patients (15.7%, HR, 0.81; 95% CI, 0.71-0.94; *P* = .005), and highest in underweight patients (28.7%, HR, 1.56; 95% CI; 1.15-2.18; *P* = .005) compared with normal weight (18.9%, reference) (Tables 2 and 4). In a multivariate cox regression model, 1-year mortality remained significantly higher in underweight (HR, 1.52; 95% CI, 1.10-2.09; *P* = .011), but in not overweight and obese patients (Table 4). Kaplan-Meier survival curves per BMI category are displayed in Figure 4.

**Table 4:** One-year outcomes according to body mass index category

Category	Unadjusted hazard ratio (95% confidence interval)	*P* value	*E* value	Adjusted hazard ratio (95% confidence interval)	*P* value	*E* value
1-y mortality						
Underweight (n = 205)	1.56 (1.15-2.18)	.005	2.06	1.52 (1.10-2.09)	.011	2.01
Normal weight (n = 3564)	Reference	–		Reference	–	
Overweight (n = 5460)	0.80 (0.71-0.91)	<.001	1.61	0.89 (0.79-1.02)	.09	1.39
Obese (n = 3152)	0.81 (0.71-0.94)	.005	1.58	0.95 (0.82-1.10)	.96	1.23
1-y stroke						
Underweight	1.00 (0.41-1.45)	.99	1.00	1.00 (0.40-2.49)	1.00	1.00
Normal weight	Reference	–		Reference	–	
Overweight	0.89 (0.67-1.18)	.43	1.50	0.94 (0.68-1.30)	.71	1.32
Obese	0.76 (0.54-1.06)	.76	1.96	0.74 (0.49-1.13)	.17	2.04

The multivariate model included age, sex, history of percutaneous coronary intervention, history of coronary artery bypass graft, diabetes mellitus, hypertension, dyslipidemia, glomerular filtration rate <30 mL/min/1.73 m^2^, valve type, mean aortic valve gradient, logistic European System for Cardiac Operative Risk Evaluation, and Society of Thoracic Surgeons Predicted Risk of Mortality.

### Other Clinical Outcomes

In hospital and 30-day stroke rates were similar across BMI groups (Table 2). Thirty-day stroke rates were comparable between centers with and without an adjudication committee.^18^ Also, 1-year stroke rates were not significantly different between BMI categories (Tables 3 and 4).

Similarly, no differences were observed for myocardial infarction and new onset atrial fibrillation (Tables 2 and 3). Major or life-threatening bleeding was less frequent in obese patients (4.4% in obese vs 5.9% in underweight, 6.3% in normal weight, and 6.0% in overweight patients; *P* = .001). Even after multivariate adjustment, bleeding incidence was lower in overweight (OR, 0.74; 95% CI, 0.59-0.94; *P* = .012) and obese patients (OR, 0.62; 95% CI, 0.46-0.82; *P* = .001). On the contrary, pacemaker implantation occurred more frequently in overweight (15.0%, OR, 1.26; 95% CI, 1.10-1.44; *P* = .001) and obese patients (14.8%, OR, 1.27; 95% CI, 1.10-1.48; *P* = .001), as opposed to normal weight and underweight patients (12.3% and 9.8%, respectively, *P* = .001). Pacemaker implantation rates remained higher after multivariate adjustment in overweight (OR, 1.30; 95% CI, 1.10-1.53; *P* = .002) and obese patients (OR, 1.30; 95% CI, 1.07-1.58; *P* = .001). Device success, as defined by VARC2,^20^ was lower in underweight patients: 89.9% compared with 94.1% in normal weight, 95.1% in overweight, and 95.7% in obesity (*P =* .001). Hospital stay was longer in underweight patients: 8 days (IQR, 6-12 days) versus 7 days (IQR, 5-11 days) in the other BMI groups (*P* < .001).

## Discussion

### Main Results

**Figure 5:**
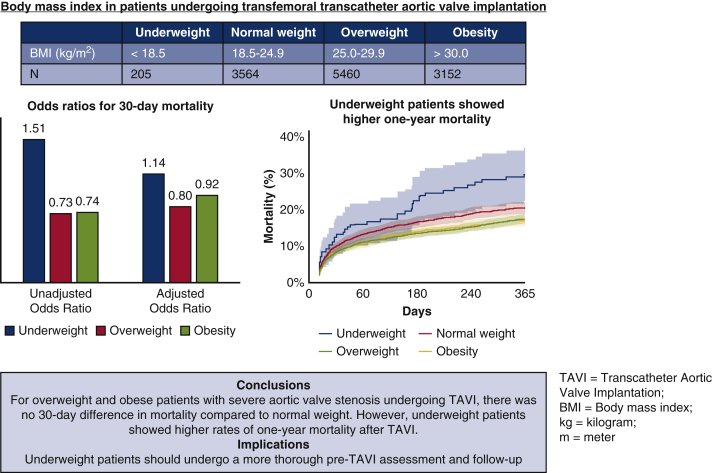
Mortality outcomes per body mass index (*BMI*) category in patients undergoing transcatheter aortic valve implantation (*TAVI*).

In this large global cohort we found that overweight and obese patients showed lower 30-day mortality compared with normal weight patients. However in multivariate analysis, there were no differences in 30-day mortality across BMI groups. Nonetheless at 1-year follow-up, underweight patients showed higher 1-year mortality rates. Stroke rates did not differ across BMI categories. Obese and overweight patients less frequently experienced major or life-threatening bleeding, but more frequently had a permanent pacemaker implanted. Figure 5 summarizes our findings. The main results are discussed by the fist author in Video 1.

### Obesity in TAVI Patients

In the present study, overweight and obese TAVI patients seem to have a prognosis benefit in univariate analysis. Previous studies showed a linear relationship between each 1 unit of BMI increase and survival.^22^ A subanalysis of the French Aortic National CoreValve and Edwards 2 Registry described better midterm survival for obese patients.^23^ Numbers of patients with extreme obesity were low but they describe a possible trend toward increased mortality for severely obese patients. In univariate analysis, we found an inverted *J*-shaped relationship in our cohort, with highest mortality rates for underweight and severely obese patients. This is consistent with Gonzalez Ferreiro and colleagues,^24^ suggesting a *J*-shaped relationship of mortality with BMI. In our cohort, the association of BMI and mortality was not significant after multivariate adjustment, which is consistent with studies of patients undergoing surgical aortic valve replacement.^15^ Despite not being an independent predictor for 30-day mortality, we studied how the outcomes are in obese patients undergoing TAVI. In clinical practice, obese patients come with their comorbidities and often metabolic syndrome, not just increased body weight. Therefore, the unadjusted results have clinical value because they represent outcomes of the total picture of obese patients undergoing TAVI.

A possible simple factor contributing to improved survival in overweight and obese patients is that these patients were younger when undergoing TAVI. Obesity can be a contraindication for cardiac surgery in the heart team decision making process. It could also be postulated that patients with obesity show symptoms of severe aortic stenosis at an earlier stage, such as dyspnea, due to the aggravating factor of excess body weight. Furthermore, they had significantly lower rates of severe kidney disease, a comorbidity strongly associated with mortality.^25^ Obese patients present a greater metabolic and physical reserve, potentiating a better tolerance for weight loss in stressful situations, such as undergoing a hospitalization for TAVI, and catabolic states, such as heart failure.^26^ Additionally, it has been demonstrated that patients with higher BMI show better adherence to guideline recommended treatment and they probably are treated more aggressively with cardioprotective drugs, conferring a better prognosis.^27^ As such, in overweight and obese patients, it looks like benefits of an increased BMI outweigh the risks, leading to better survival rates. This relationship seems to invert for extremely obese patients, where the comorbidities associated to their condition mitigate the benefits of increased body weight.

### Low BMI: A Surrogate for Frailty

Patients with a BMI <18.5 kg/m^2^ undergoing TAVI have been less well studied in previous analyses due to small sample sizes.^17^^,^^19^^,^^20^ In our cohort, there were 205 underweight patients, which to our knowledge corresponds to the largest number observed in this category. These patients presented a poor prognosis after TAVI, with mortality rates as high as 8.4% in-hospital, 9.8% at 30-day follow-up, and 28.7% at 1-year follow-up. Even after multivariate adjustment, 1-year mortality was 1.5 times higher than normal weight patients. Low BMI is strongly associated with frailty, cardiac cachexia, and overall sicker patients.^15^^,^^16^ Taking these findings together, we hypothesize that a BMI <18.5 kg/m^2^ might be used in TAVI patients as a simple frailty discriminator. The possible lack of benefit of this invasive procedure for these very frail patients should be carefully analyzed. The difference in mortality in underweight patients was statistically significant at 1-year follow-up, but not at 30 days. This difference points toward the fact that higher 1-year mortality can be explained by deaths from other causes. These 1-year deaths may not be related to the TAVI procedure itself, but rather to overall frailty. Prior to TAVI, extensive assessment, including adjustments to improve frailty is warranted. For example, patients can be assessed by a geriatrician and nutritional status can be optimized to improve clinical outcomes. Nevertheless, TAVI should be carefully considered in patients with limited life expectancy because of comorbidities or overall frailty. Low BMI can be considered a red flag, signaling patients at increased risk of poor prognosis after TAVI. Device success, according to VARC2,^20^ was less often achieved in underweight patients. This finding supports the hypothesis of frailty in underweight patients.

### Other Clinical Outcomes

In this analysis, BMI was not associated with rates of stroke. This is in line with previous studies.^23^^,^^24^ In a recent article presented by the CENTER collaboration, BMI was not an independent predictor of stroke.^18^ Overweight and obese patients less frequently experienced major or life-threatening bleeding, even after multivariate adjustment. This is consistent with other studies reporting higher bleeding incidence in underweight and lower bleeding incidence in obese patients after TAVI,^23^ cardiac valve surgery,^14^ and percutaneous coronary intervention.^17^

### Limitations

This study is a retrospective analysis, with all its inherent limitations. Nonetheless, it represents a global and real-world patient population of subjects undergoing transfemoral TAVI over the past decade. Secondly, although BMI is the widest used parameter to investigate the influence of body weight on outcomes, it does not give detailed information about body composition, such as the percentage of lean body mass and the fat distribution pattern (central vs peripheral). Thirdly, we recorded BMI at a single point in time (immediately before the TAVI procedure). We did not include data about weight gain or weight loss after TAVI.

### Impact on Daily Practice

Thirty-day mortality was not different across BMI groups. However, 1-year mortality was higher in underweight patients. Therefore efficiency of TAVI treatment should be carefully considered in this subgroup. Moreover, interventions to improve frailty status before TAVI and a closer follow-up after TAVI are warranted.

## Conclusions

In more than 12,000 patients undergoing transfemoral TAVI, overweight and obese patients showed lower 30-day mortality as compared to patients with normal weight. However, no differences in 30-day mortality between BMI groups were found after multivariate adjustment. At 1-year follow-up, underweight was an independent predictor of higher mortality after TAVI.

### Conflict of Interest Statement

Dr Regueiro is a proctor for Abbott Vascular. All other authors reported no conflicts of interest.

The *Journal* policy requires editors and reviewers to disclose conflicts of interest and to decline handling or reviewing manuscripts for which they may have a conflict of interest. The editors and reviewers of this article have no conflicts of interest.

## Figures and Tables

**Table E1:** Proportion of missing data per body mass index category

Variable	BMI category					
	<18.5 kg/m^2^ (n = 205)	18.5-24.9 kg/m^2^ (n = 3564)	25-29.9 kg/m^2^ (n = 5460)	30.0-34.9 kg/m^2^ (n = 2257)	35-39.9 kg/m^2^ (n = 658)	≥40 kg/m^2^ (n = 237)
Procedural						
Conversion to open heart surgery	3 (1.3)	200 (5.6)	636 (11.6)	194 (8.6)	28 (4.3)	6 (3.3)
During hospital admission						
Mortality	2 (1.0)	40 (1.1)	89 (1.6)	55 (2.4)	13 (2.0)	6 (3.3)
Stroke	0 (0)	29 (0.8)	87 (1.5)	61 (2.7)	18 (2.3)	2 (2.9)
Myocardial infarction	18 (8.8)	227 (6.4)	310 (5.7)	133 (5.9)	29 (4.4)	11 (4.6)
Major or life-threatening bleeding	18 (8.8)	361 (10.1)	496 (9.1)	193 (8.6)	58 (8.8)	18 (7.6)
New-onset atrial fibrillation	55 (26.8)	1284 (36.0)	2427 (44.4)	1135 (50.3)	360 (54.7)	123 (51.9)
Permanent pacemaker implantation	4 (19.5)	338 (5.9)	615 (11.2)	188 (8.3)	22 (3.3)	7 (3.0)
At 30 d						
Mortality	18 (8.8)	296 (8.3)	521 (9.5)	215 (9.6)	16 (10.4)	23 (9.7)
Stroke	18 (8.8)	297 (8.3)	528 (9.7)	216 (9.6)	16 (10.4)	23 (9.7)
At 1 y						
Mortality	62 (30.2)	1139 (31.9)	1984 (36.3)	790 (35.0)	209 (31.7)	76 (32.1)
Stroke	60 (29.3)	1097 (30.8)	1943 (35.5)	781 (34.6)	207 (31.4)	76 (32.1)

Values are presented as n (%). *BMI*, Body mass index.

**Table E2:** Thirty-day mortality and stroke rates according to body mass index category in patients with balloon-expandable and self-expandable valves

Variable	BMI category					
	<18.5 kg/m^2^ (n = 205)	18.5-24.9 kg/m^2^ (n = 3564)	25-29.9 kg/m^2^ (n = 5460)	30.0-34.9 kg/m^2^ (n = 2257)	35-39.9 kg/m^2^ (n = 658)	≥40 kg/m^2^ (n = 237)
Allocation						
Balloon-expandable	96 (46.8)	1851 (51.9)	2785 (51.0)	1099 (48.7)	291 (44.2)	117 (49.4)
Self-expandable	109 (53.2)	1713 (48.1)	2675 (49.0)	1158 (51.3)	367 (55.8)	120 (50.6)
30-d mortality						
Balloon-expandable	9 (10.6)	115 (7.0)	118 (4.8)	39 (4.1)	17 (7.0)	8 (7.7)
Self-expandable	10 (9.7)	111 (6.9)	137 (5.5)	55 (5.1)	22 (6.3)	8 (7.3)
*P* value	.84	.96	.29	.27	.74	.91
30-d stroke						
Balloon-expandable	3 (3.5)	48 (2.9)	58 (2.4)	17 (1.8)	7 (2.9)	7 (2.9)
Self-expandable	2 (2.0)	48 (3.0)	67 (2.7)	26 (2.4)	9 (2.6)	2 (1.8)
*P* value	.51	.88	.48	.31	.82	.59

Values are presented as n (%). *BMI*, Body mass index.

## Supplementary Data

Video 1 The first author briefly discusses the main results of this study. Thirty-day mortality after transcatheter aortic valve implantation was lowest in overweight and obese patients. However, after multivariate adjustment, 30-day mortality was not different across body mass index groups. Nevertheless, underweight patients showed higher 1-year mortality. Video available at: https://www.jtcvs.org/article/S2666-2736(21)00069-3/fulltext.
