# Supplementary figures and images for: Impact of body mass index on outcomes in patients undergoing transfemoral transcatheter aortic valve implantation
Source: JTCVS Open. 2021 Mar 23;6:26–36. doi: 10.1016/j.xjon.2021.03.012 (PMC9390374; doi:10.1016/j.xjon.2021.03.012)

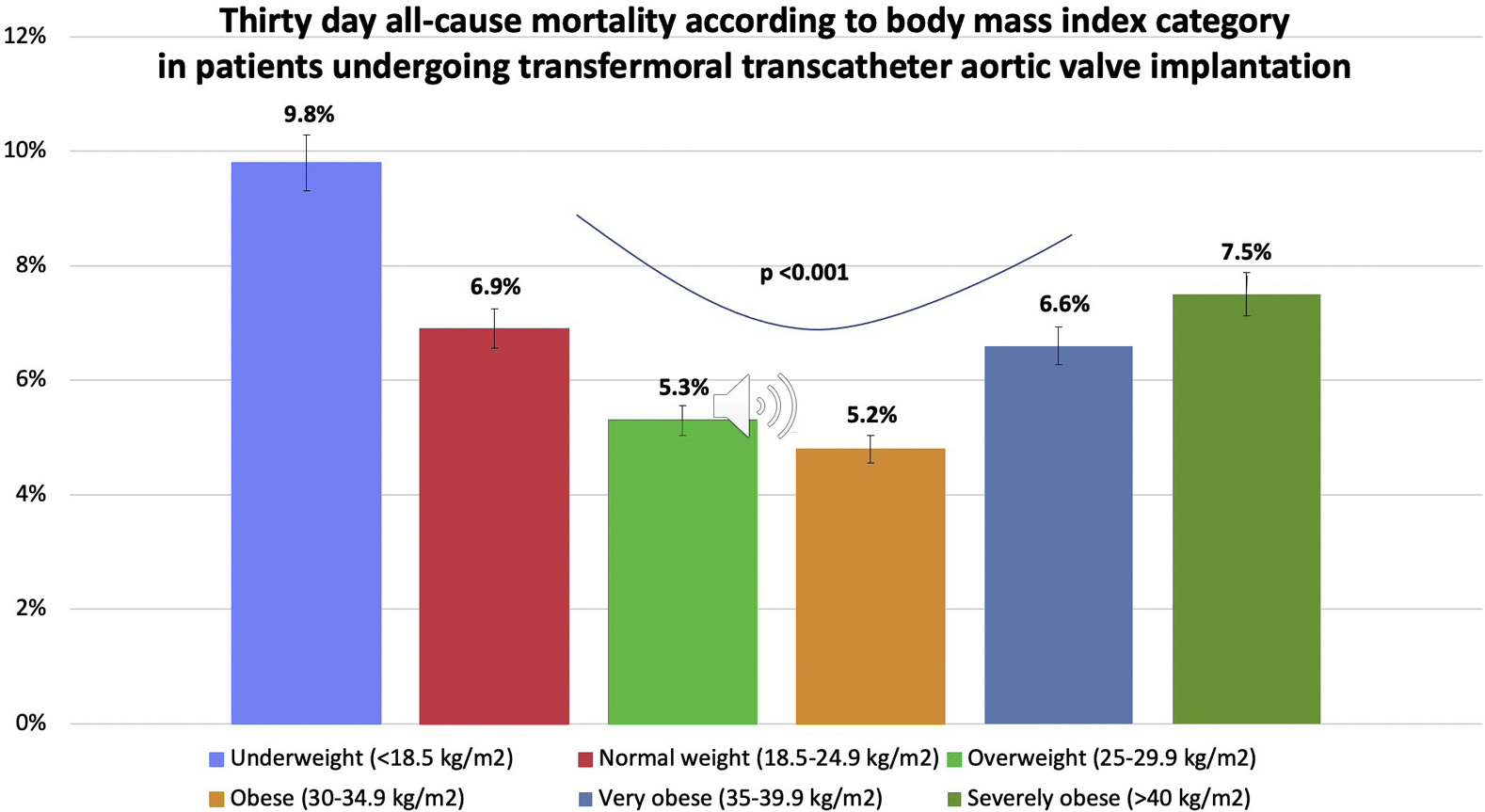

Supplement: Video 1 — The first author briefly discusses the main results of this study. Thirty-day mortality after transcatheter aortic valve implantation was lowest in overweight and obese patients. However, after multivariate adjustment, 30-day mortality was not different across body mass index groups. Nevertheless, underweight patients showed higher 1-year mortality. Video available at: https://www.jtcvs.org/article/S2666-2736(21)00069-3/fulltext. [file fx3.jpg]
